# Author Correction: Lead federated neuromorphic learning for wireless edge artificial intelligence

**DOI:** 10.1038/s41467-022-32602-8

**Published:** 2022-08-17

**Authors:** Helin Yang, Kwok-Yan Lam, Liang Xiao, Zehui Xiong, Hao Hu, Dusit Niyato, H. Vincent Poor

**Affiliations:** 1 Department of Information and Communication Engineering, School of Informatics, Xiamen University, Xiamen, China; 2 Strategic Centre for Research in Privacy-Preserving Technologies and Systems, Nanyang Technological University, Singapore, Singapore; 3 School of Computer Science and Engineering, Nanyang Technological University, Singapore, Singapore; 4 Pillar of Information Systems Technology and Design, Singapore University of Technology and Design, Singapore, Singapore; 5 Department of Electrical and Electronic Engineering, Nanyang Technological University, Singapore, Singapore; 6 Department of Electrical and Computer Engineering, Princeton University, Princeton, NJ USA

**Keywords:** Computer science, Electrical and electronic engineering, Computational science

**Correction to:** *Nature Communications* 10.1038/s41467-022-32020-w, published online 25 July 2022

The original version of this Article contained an error in the text (in the first paragraph), which was previously incorrectly given as “Such capability cannot only facilitate data privacy preservation but also reduce data traffic and network latency”. The correct version states “Such capability not only facilitates data privacy preservation but also reduces data traffic and network latency”.

The original version of this Article contained an error in the text (Page 2), which was previously incorrectly given as “Schematic of a social leaning network, where each human uses five sensory organs to interact the outside environment”. The correct version states “Schematic of a social learning network, where each human uses five sensory organs to interact with the outside environment”.

The original version of this Article contained an error in the text (Page 2), which was previously incorrectly given as “Inspired by the collaborative human learning system (a) a federated”. The correct version states “Inspired by the collaborative learning system in (a) a federated”.

The original version of this Article contained an error in the text (Page 9), which was previously incorrectly given as “with a leader to performance model aggregation”. The correct version states “with a leader to perform model aggregation”.

The original version of this Article contained an error in the Acknowledgements, which was previously incorrectly given as “Singapore and Infocomm Media Development Authority under its Future Communications Research & Development Programme”. The correct version states “the National Research Foundation, Singapore and Infocomm Media Development Authority under its Future Communications Research & Development Programme”.

These have been corrected in both the PDF and HTML versions of the Article.

