# Supplementary material for: Lead federated neuromorphic learning for wireless edge artificial intelligence
Source: Nat Commun. 2022 Jul 25;13:4269. doi: 10.1038/s41467-022-32020-w (PMC9314401; doi:10.1038/s41467-022-32020-w)
Supplement: Supplementary file 1 — Supplementary Information [file 41467_2022_32020_MOESM1_ESM.pdf]

Supplementary information (SI):

Lead federated neuromorphic learning for wireless edge artificial intelligence

Helin Yang, Kwok-Yan Lam*, Liang Xiao, Zehui Xiong, Hao Hu,
Dusit Niyato and H. Vincent Poor

*Corresponding author. E-mail: kwokyan.lam@ntu.edu.sg

Supplementary Note 1: Leader election for federated learning

The leader election plays an important role in federated model aggregation performance for edge AI. A leader with high computation and communication capabilities can speed up the federated aggregation process and reduce the overall training latency, whereas the federated aggregation latency will be negatively impacted if the network elects a leader with low computation and communication capabilities. For example, as illustrated in Supplementary Fig. 1a, there are three leader election scenarios in terms of communication capability evaluation. We consider the communication capability as an example for leader election, and assume that other metrics (e.g., computation and energy supply capabilities) are equal for all devices. Note that, as the communication distance increases, the data rate deceases[1-3], thus leading to an increase in packet transmission latency. For Scenario 1 or Scenario 2, the system does not take the communication distance (wireless communication link quality) into account but randomly elects of the devices as the leader to perform model aggregation. In this case, as the elected leader (device 5 or device 6) is located at the edge, the followers (e.g., device 2) are located at the opposite corner have a greater communication distance, resulting in a limited communication data rate and increasing the data packet transmission latency. The leader needs to wait for the last follower to upload its local model parameters before performing model aggregation, which directly increases the overall processing delay. On the contrary, this issue can be effectively addressed by considering the communication capability into the leader election, as shown in Scenario 3 (Supplementary Fig. 1a). Here, the elected leader is located at the center of the edge devices, and the communication distance (wireless communication link quality) from followers to the leader are relatively balanced, and thus this avoid the situation in which the communication distance between any edge follower and the leader is extremely far. In this context, taking

the device's communication capacity into account for the leader election can greatly improve the federated model aggregation in terms of lower latency.

The flow chart for leader election is shown in Supplementary Fig. 1b. Firstly, each participating edge device calculates its weighted score based on three metrics, i.e., computation, communication and energy supply capabilities, where the weighted score is given by

$$Weighted\ Score = Communication\ capability + Computation\ capability + Energy\ supply\ capability. \quad (1)$$

Then, each candidate advertises its score for leader election. The device with the highest score is elected as the leader to perform federated model aggregation.

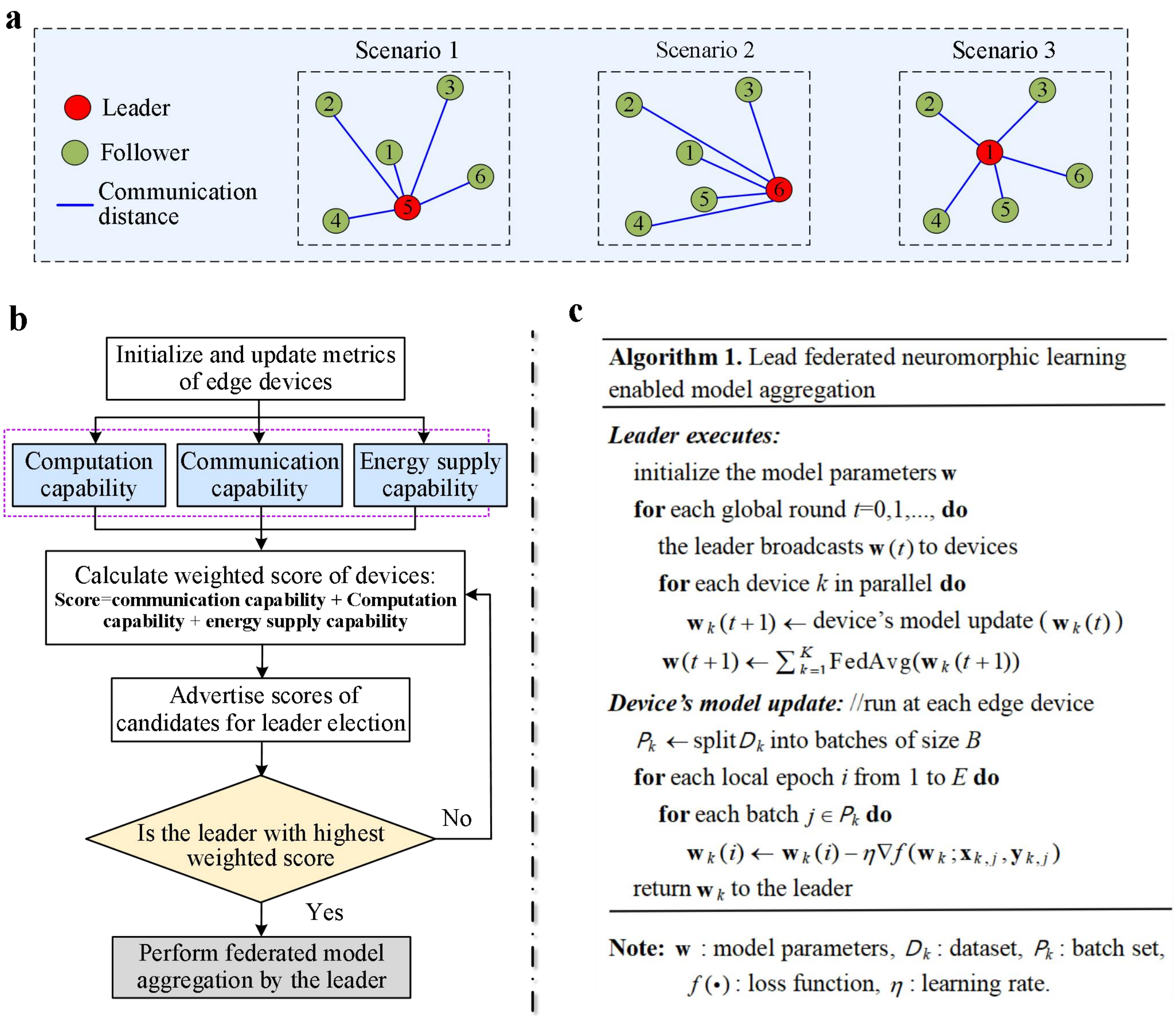

Supplementary Fig. 1: Leader election and federated model aggregation process. a, An example of the leader election in three scenarios in terms of communication capability. **b**, Flow diagram of proposed leader election protocol. **c**, Algorithm of the LFNL enabled federated model aggregation process.

Once the leader is elected, federated learning can be implemented which is shown in Supplementary Fig. 1c. Firstly, the leader broadcasts the initialized model $\mathbf{w}$ to all followers. Each device trains its own local model independently and in parallel based on its dataset, and then uploads its local model parameters $\mathbf{w}_k$ to the leader. The leader collects local model parameters ($\mathbf{w}_1, \mathbf{w}_2, \mathbf{w}_3, ..., \mathbf{w}_K$) to perform model aggregation before broadcasting the updated global parameters $\mathbf{w}$ to followers for the next round of local training. The exchange of local and global parameters repeats until convergence. Here, the federated averaging method is used for model aggregation[1].

As mentioned earlier, leader election aims to accelerate the federated training process. Thus, both the computation and communication times are also essential metrics to be optimized. Before evaluating the performance of the leader election scheme, we introduce the definitions of the computation and communication times in federated learning systems as follows[2,3].

The computation time T_k of the k-th device mainly depends on its training dataset size $|D_k|$ and the computation capability. In each global epoch, the local computation time of device k can be calculated as

$$T_k^{\text{local}} = E_k C_k |D_k| / f_k , \tag{2}$$

where C_k (cycles/bit) denotes the number of Central Processing Unit (CPU) cycles needed for computing with a single data sample, E_k is the number of local training epochs and f_k is the CPU frequency of device k. For the leader, the computation time per global epoch can be calculated as

$$T_{\text{test}} = C_{\text{leader}} |D_{\text{test}}| / f_{\text{leader}} , \tag{3}$$

where C_{test} is the number of CPU cycles needed for computing with a single data sample, $|D_{\text{test}}|$ is the testing dataset size of its set D_{test}, and f_{leader} is the CPU frequency of the elected leader.

In each global epoch, each device needs to upload its local training model parameters to the leader for model aggregation, and the leader also broadcasts the updated global model parameters to all participating followers for the next training process. During this process, the communication time of one model upload or broadcast between the leader and the k-th follower can be expressed as

$$T_k^{\text{com}} = |D_{\text{model}}| / R_k , \tag{4}$$

where $|D_{\text{model}}|$ is the size of the set of model parameters D_{model}, and R_k is the the achievable data rate between the leader and follower k, which is given by

$$R_k = B\log_2(1+\frac{Ph_k}{\delta^2}), \quad (5)$$

where B is the wireless transmission bandwidth, P is the transmission power, h_k is the channel gain between the leader and device k, and δ^2 is the background Gaussian noise power. We assume that all devices have the same bandwidth and transmission power.

Overall, the total training time depends on communication and computation times over a number of global epochs I. Here, we use the synchronized updates property in the federated system[4], where the leader begins to aggregate the global model until all followers upload their local models. Thus, the overall training time is given by

$$T = I\left(\max_k T_k^{\text{local}} + \max_k(2\times T_k^{\text{com}}) + T_{\text{test}}\right). \quad (6)$$

Note that in equation (6), the model parameters are required to be uploaded and downloaded, and thus the communication time is $2\times T_k^{\text{com}}$ per global epoch.

We consider a single-cell disc-shaped wireless network with a radius of 60 meters. Six devices are randomly located in the cell. The path loss between one device and another device is $h = d^{-4}$ [5,6] with d being the communication distance in meters. We assume that each device has B=0.5 MHz of bandwidth for uploading and downloading model parameters. The transmission power P and background Gaussian noise power δ^2 are set as 50 mW and -100 dBm, respectively. The computation frequency of devices is uniformly set from the set [0.5, 1.0, 1.5] GHz. The parameter C is uniformly distributed in [50, 100] cycles/bit for all devices. Each device has $|D|=1553$ Kbits of training samples, $|D_{\text{model}}|=2296$ Kbits of model parameters, and the testing dataset size is $|D_{\text{test}}|=1170$ Kbits. In the benchmark SNNs, the number of nodes of the input layer is 1728, the number of neuron nodes of one hidden layer is 300, and the number of nodes of the output layer is 3. The traffic image dataset[7,8] is used for performance elevation, in total, 872 images are used with three classes, including 160 bicycle images, 205 car images, and 507 traffic light images. 80% of the images are used for training, and the remaining 20% are used for validation and testing. The training dataset is divided into six small parts for six devices, and we run the experiments on a laptop.

Here we provide the performance comparisons between the proposed leader election scheme and the random leader election scheme. Supplementary Fig.2 shows the obtained convergence speed and test accuracy throughout the federated training time for the two schemes. Overall, the proposed LFNL method

considers computation and communications aspects in the leader election that results in the convergence speed enhancements supporting real-time edge computing deployments. In particularly, we find that LFNL obtains the faster convergence speed while maintaining the higher test accuracy via training time slots. For example, as illustrated in Supplementary Fig.2a, when the validation loss is 0.025, the training completion times of the leader election scheme and the random leader election scheme are 20.6s and 34.3s, respectively, significantly reducing the overall training time by nearly 66.5%. The reason for this lies in the election of a leader with high computation and communication capabilities to perform federated model aggregation, which substantially reduces computation and communication times. Hence, LFNL with leader election is valuable in accelerating the federated learning process on edge devices.

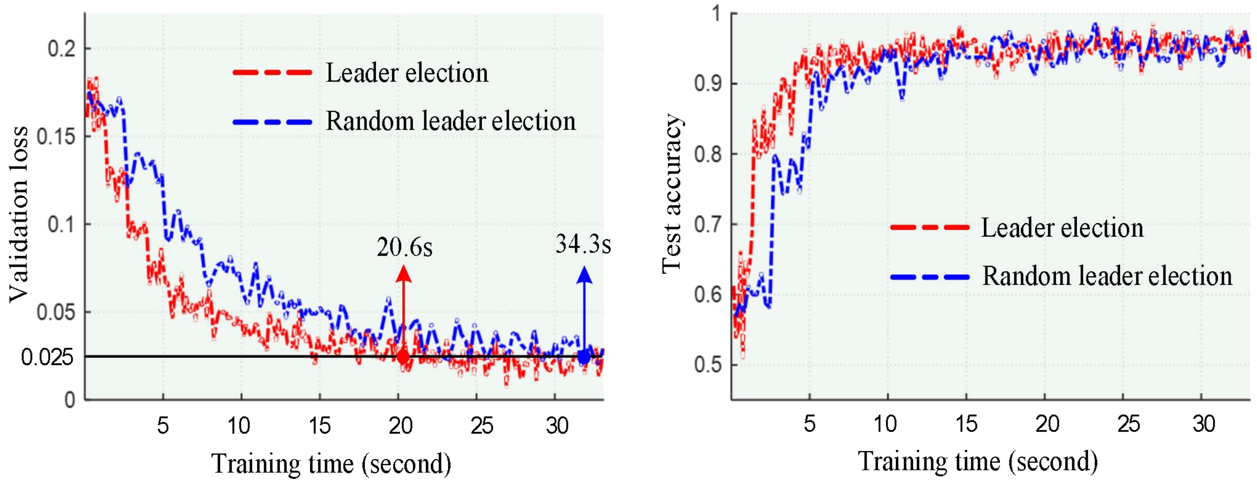

Supplementary Fig. 2: Performance evaluation of LFNL-based leader election on traffic image dataset[7,8]. a, Validation loss curves via training time for the leader election and random leader election schemes. **b**, Test accuracy via training time for the leader election and random leader election schemes.

Supplementary Note 2: Learning with more edge devices on the traffic sound dataset

As described in the main manuscript, AI performs well when the training data is sufficient[9,10], for example, the test accuracy values of device 1 and device 2 (Fig. 2h in the main manuscript) are quite good. To demonstrate the scalability and robustness of LFNL, we divide the training samples into six parts for six devices with each having a smaller training dataset, the dataset distribution at the six devices is equally set as 13.3%. As depicted in Supplementary Figs. 3a,b, not only are the test accuracy values of

the six locally training devices significantly lower than that of LFNL, but also their test accuracy values fluctuate more frequently due to insufficient training samples. This is because the insufficient training sound samples enable local learning to overfit quickly and lead to an unstable training model. In this scenario, LFNL still achieves the comparable test accuracy of 92.4% to the centralized learning method (shown in Fig. 2l with yellow color box in the main manuscript).

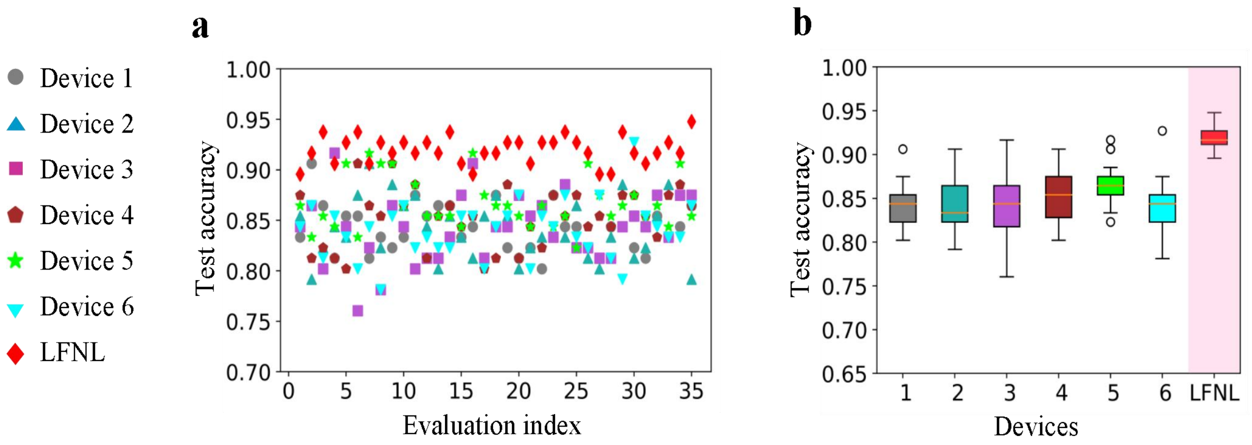

Supplementary Fig. 3: LFNL for audio recognition with scenario that dividing the training dateset for six edge devices training data divided among six edge devices. a, 35 independent test accuracy evaluations for six locally training devices and LFNL after training. The dataset distributions for the six devices are equally set as 13.3%. **b**, Box plots show test accuracies for the 35 independent evaluations of Supplementary Fig. 3a.

Supplementary Note 3: Learning under poisoning model attack on the traffic sound dataset

In large-scale federated learning systems, performance degradation may arise due to malicious or low-quality participants[11], where poisoning or low-quality model parameters hamper the global accuracy of jointly trained model by uploading malicious or low-quality inputs of the jointly trained model. Fortunately, as reported[12-14], various poisoning model detection algorithms have been proposed to defend against poisoning attacks, such as generative adversarial networks-based FL[12] and participant selection[3]. To demonstrate the robustness of LFNL to such issues, we also examine it in the presence of poisoning or low-quality models.

We consider an experimental scenario (Supplementary Fig. 4a) in which the traffic sound dateset[15] is divided into six small parts for six edge devices, where one of six devices acts as a malicious participant with poisoning or a low-quality model and the others are normal. Here, we compared the following leaning methods: 1. The normal federated learning system with no positioning model attack and all edge devices are normal, denoted by *no positioning model attack*. 2. One malicious edge device joins the federated learning system and sends its poisoning model parameters to degrade the global training accuracy. All devices are decentralized edge devices and no leader detects the poisoning model, but rather all devices are selected for model aggregation, denoted by *decentralized FNL(DFNL) without selection.* 3. The leader detects the poisoning model and selects the normal devices for model aggregation, denoted by *LFNL with selection.*

In the experiments presented in Supplementary Fig. 4a, due to the poisoning model attack during the federated model aggregation process, the validation loss curve of the method (DFNL without selection) exhibits unstable training. Interestingly, its validation loss values increase as the number of training epochs increases, leading to a low test accuracy of 80.3% (Supplementary Figs. 4b,c). On the contrary, as indicated by a lower loss and higher test accuracy in Supplementary Fig. 4 (red color), LFNL with selection defends against the poisoning model attack and significantly outperforms DFNL without selection. The reason is that the proposed method (LFNL with selection) enables the system to select a leader to detect the malicious device, and schedules normal devices to participate in the federated model aggregation. Thus, its loss curve is smoother and its classification accuracy is higher than those of DFNL without selection. In these results, we also find that the performance of LFNL with selection is approximately equivalent to the system with no poisoning attack. This gives us confidence that it can effectively defend against the poisoning model attack during federated model aggregation for edge AI.

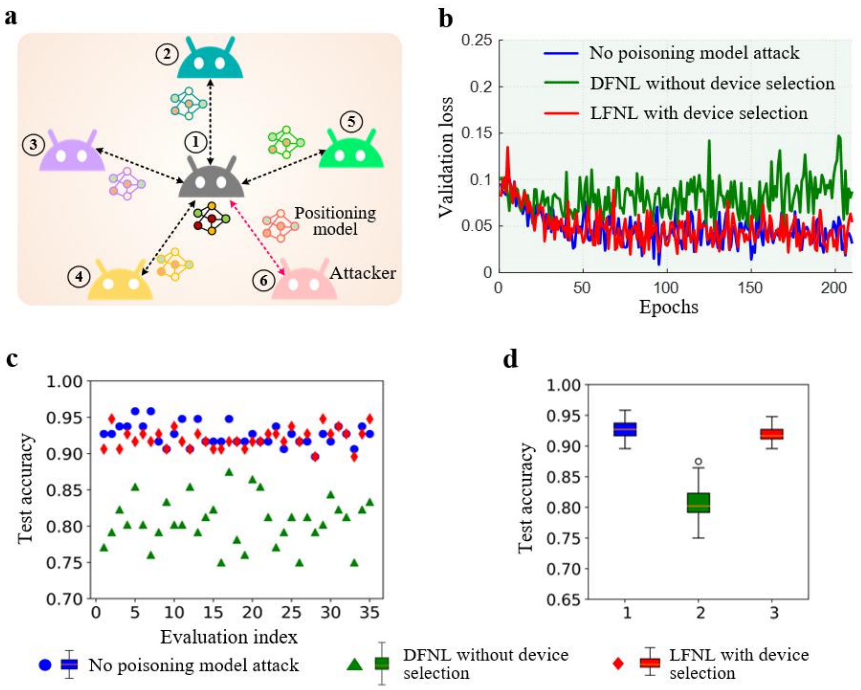

Supplementary Fig. 4: Performance evaluation under a poisoning attack on the traffic sound dataset. a, Scenario that one of six edge devices is a malicious participant with poisoning or a low-quality model to attack the federated learning model, while other devices are normal. The dataset distributions for the six devices are equally set as 13.3%. **b**, Validation loss curves for the three learning methods. **c**, 35 independent test accuracy evaluations for three learning methods after training. **d**, Box plots show test accuracies for the 35 independent test evaluations of Supplementary Fig. 4c.

Supplementary Note 4: Learning against image rotation on the traffic image dataset

In practical applications, collected images may not all be at the same orientation, but rather may be rotated at random angles. In this section, we demonstrate the classification robustness of LFNL on the traffic image dataset[7,8] in the presence of image rotation (Supplementary Fig. 5a). In the benchmark LFNL, the SNN has 1728-2500-3 neurons (input-hidden-label layer). For the traffic image dataset[7,8], in total, 872 images were used with three classes, including 160 bicycle images, 205 car images, and 507 traffic light images. 80% of the images are used for training, and remaining 25% are used for validation and testing.

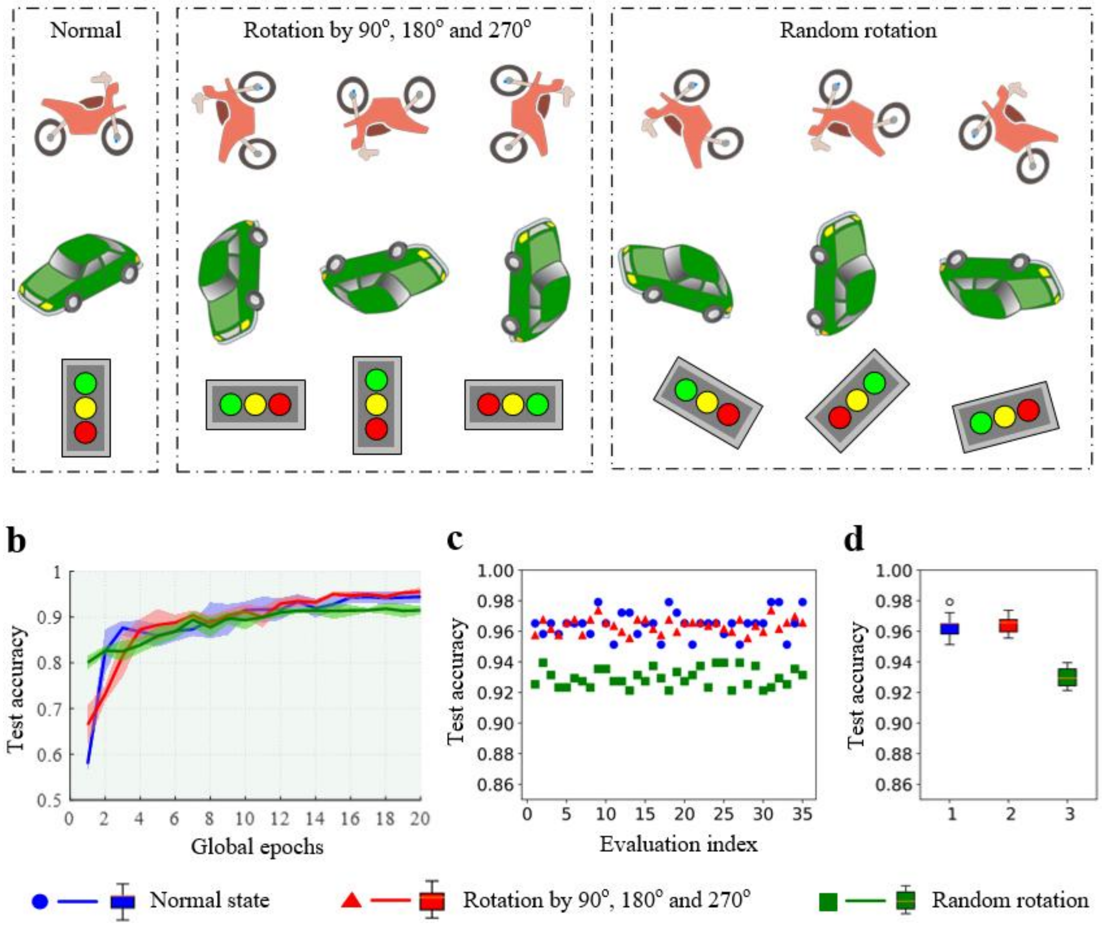

Supplementary Fig. 5: Classification evaluation of LFNL on the traffic image dataset under image rotation. a, Three scenarios in which traffic images are rotated, i.e., normal state with no image rotation, image rotation by 90° , 180°, and 270°, as well as image rotation with random angles. **b**, Validation loss curves for the three scenarios of Supplementary Fig. 5a. **c**, 35 independent test accuracy evaluations for three learning methods after training. **d**, Box plots show test accuracies for the 35 independent evaluations of Supplementary Fig. 5c.

As illustrated in Supplementary Fig. 5b, LFNL with image rotation (90° , 180°, and 270°) achieves a similar test accuracy to the normal state with no rotation under different numbers of global training epochs, and the mean classification accuracy reaches 96.20% for these two scenarios (Supplementary Figs. 5c,d). The overall test accuracy of LFNL with random image rotation is just slightly lower than that of the normal state with no rotation via each global training epoch (Supplementary Fig. 5b), still achieving a test accuracy of 93.40% (Supplementary Figs. 5c,d). These results support the conclusion that the proposed LFNL method can robustly recognize/classify images even though the images are placed in different rotation angles.

Supplementary Note 5: Learning with more edge devices on MNIST and TIDIGITS datasets

To showcase LFNL's scalability and flexibility in classifying images and speech signals on larger and higher-dimensional datasets, we provide an additional case investigation where MNIST[16] and TIDIGITS[17] datasets are divided into smaller subsets for use on edge devices. In particular, 13.3% of these datasets is distributed to each of six devices.

As plotted in Supplementary Figs. 6a,b, we find that the overall test accuracy of the six locally training devices can maintain a high level (around 95%), which is slight lower than that of three locally training devices (Figs. 6a,b in the main manuscript). The reason lies in the fact that even though the 60000 training image of MNIST dataset is equally divided into six small parts for six devices, each locally training device still has sufficient samples (each has 10000 samples) to train a reliable classifier.

However, when we test the classification performance on TIDIGITS dataset by dividing it into six small parts for six edge devices, the overall test accuracy values of the six locally training devices substantially decline (Supplementary Figs. 6c,d). Moreover, the test accuracy values of the six locally training devices fluctuate more frequently than that of LFNL. The reason is that the number of training samples of each device is 660, which is significant less than that of the MNIST dataset. Therefore, local learning with insufficient training samples overfits quickly and leads to an unstable training model. On the contrary, LFNL overcomes this local overfitting issue and significantly outperforms the locally training devices (Supplementary Figs. 6c-e). It is worth noting that even if the training dataset is divided into more smaller parts for edges devices, the LFNL results still does not deteriorate (Supplementary Figs. 6c-e).

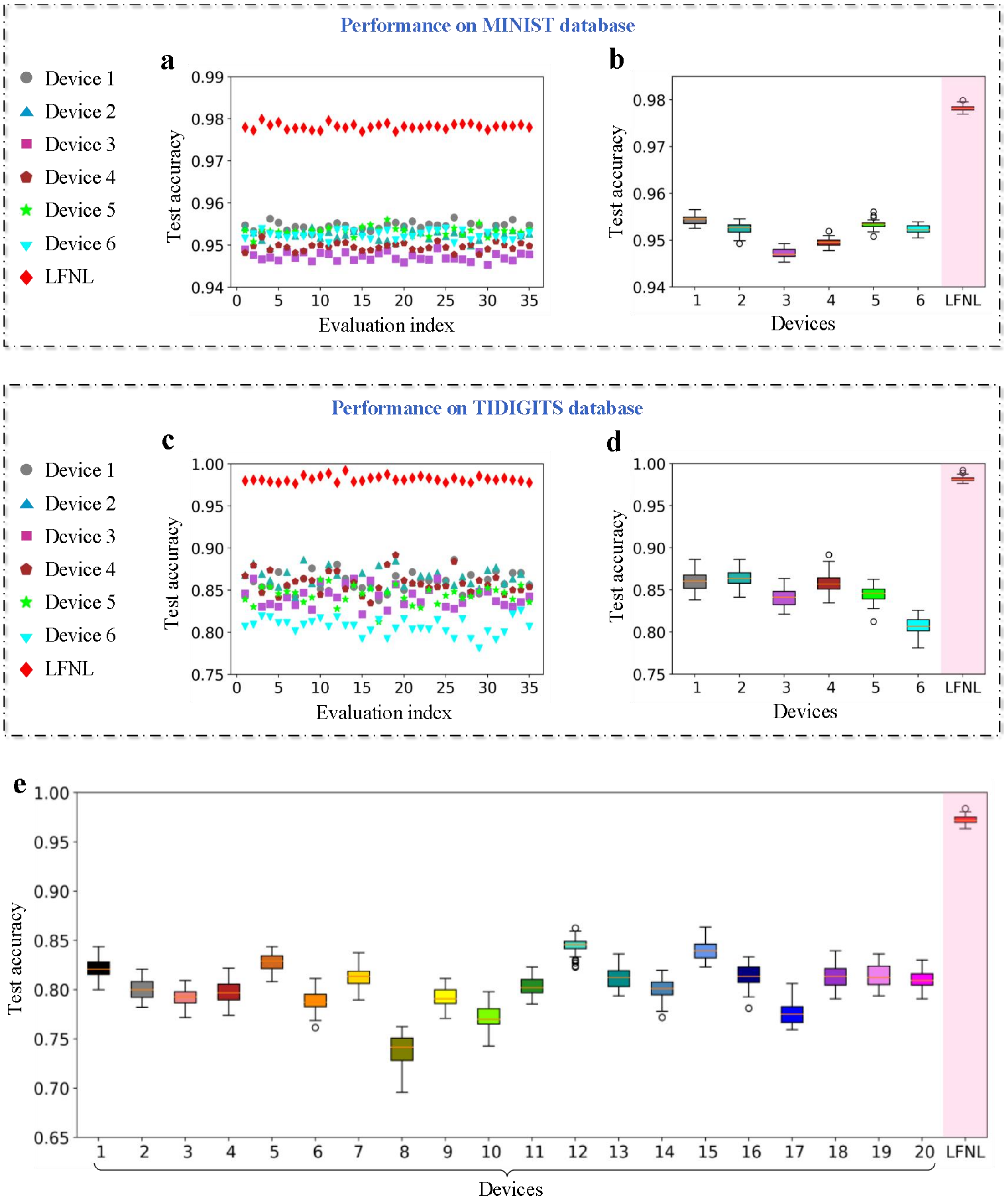

Supplementary Fig. 6: Scenario of dividing the MNIST and TIDIGITS datasets onto six edge devices. **a**, Evaluation of test accuracy for six edge devices and LFNL over 35 independent runs on the MNIST dataset. The dataset distributions for the six devices are equally set as 13.3%. **b**, Box plots show test accuracies for the 35 independent test evaluations of Supplementary Fig. 6a. **c**, Evaluation of test accuracy for six edge devices and LFNL over 35 independent runs on the TIDIGITS dataset. The dataset distributions for the six devices are equally set as 13.3%. **d**, Box plots show test accuracies for the 35 independent evaluations of Supplementary Fig. 6c. **e**, Box plots show test accuracies for twenty small edge devices and LFNL over 35 independent runs on the TIDIGITS dataset. The dataset distributions for the twenty devices are equally set as 4%.

Supplementary Figs. 7a-e show the experimental results for different learning methods on the MNIST dataset with 784-500-10 neurons (input-hidden-label layer). As observed in Supplementary Figs. 7a,b, the test accuracies of the three locally training devices are more than 92%, because the three devices have sufficient training samples (25000, 25000 and 5000 samples). By using LFNL, the test accuracy of device 3 can be significantly improved from 92.3% to 97.5%. The confusion matrix of FLNL for the test data set after training is depicted in Supplementary Fig. 7c, in which the classification accuracy of each class is more than 96%. Supplementary Figs. 7d,e capture the test accuracy and data traffic comparisons. The test accuracy of CNL is slightly higher than that of the other three methods (Supplementary Fig. 7d), but the local dataset of device has to be shared which creates greater data traffic (Supplementary Fig. 7e) and compromises private information. Although TNL has lower traffic size than that of FLNL (Supplementary Fig. 7e), it needs longer training latency as it does not use parallel training.

We further evaluated the classification capability of LFNL on the eleven-class speech TIDIGITS dataset, where Supplementary Figs. 7f-j indicate the results for learning networks with 1640-1000-11 neurons (input-hidden-label layer). Owing to sufficient training samples at three locally training devices, an approximate test accuracy of 95% at the three devices can be achieved (Supplementary Figs. 7f,g), but the performance can be enhanced to 97.1% by using LFNL. Notably, as shown in Supplementary Fig. 6i, both CNL and TNL obtain slightly higher test accuracy than those of CFNL and LFNL. However, CFNL and LFNL require 2.5× longer training latency than that of CFNL and LFNL (Supplementary Fig. 6j) as they train sequentially rather than in parallel, which may not be suitable for real-time edge AI.

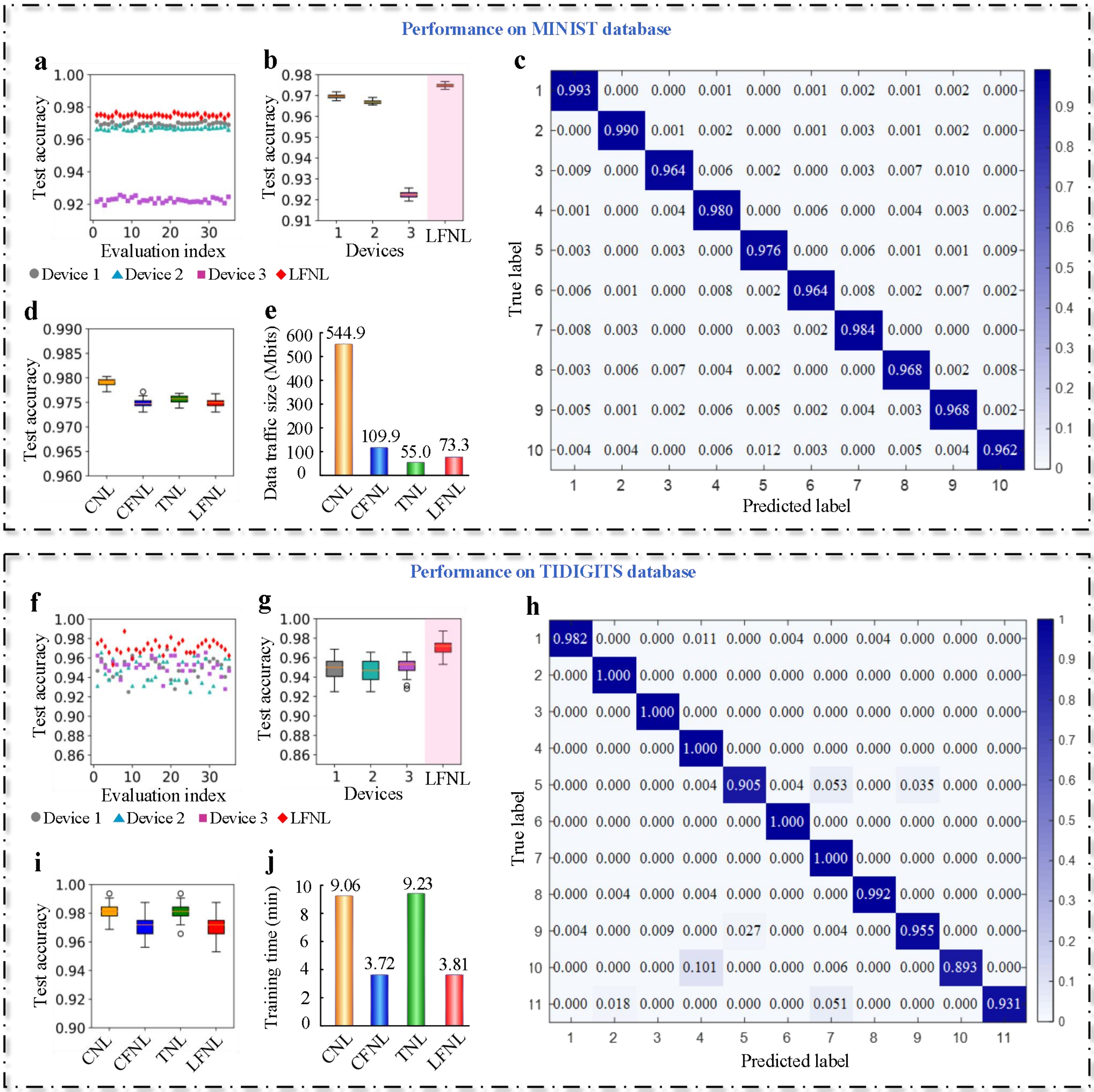

Supplementary Fig. 7: Classification evaluation on MNIST and TIDIGITS datasets. **a**, 35 independent test accuracy evaluations for three locally training devices and LFNL after training on the MNIST dataset. The dataset distributions for the three devices are accordingly set as 38.8%, 38.5% and 7.7%, respectively. **b**, Box plots show test accuracies for 35 independent test evaluations of Fig. 6a. **c**, Confusion matrix for the test set in LFNL after training. **d**, **e**, Test accuracy and data traffic size performance comparisons for different methods on the MNIST dataset, respectively. **f**, **g**, Test accuracies evaluation and box plots show test accuracy for three locally training devices and LFNL on the TIDIGITS dataset after 35 independent experimental runs. The dataset distribution for the three devices are accordingly set as 37.7%, 37.7% and 6.2%, respectively. **h**, Confusion matrix for the test set in LFNL after training. **i**, **j**, Test accuracy and training latency performance comparisons for different methods on the TIDIGITS dataset, respectively.

Supplementary Fig. 8 shows the performance evaluations of the two methods on the MNIST and TIDIGITS datasets with small training data sizes, where we use a 20% training data size for the MNIST dataset and a 30% training data size for the TIDIGITS dataset. The training data is evenly divided into three parts for the three edge devices. From Supplementary Fig. 8, we can observe that the classification accuracy of LFNL suffers a slight loss (up to 1 % loss) compared to CNL, but it significantly achieves a lower traffic size and training latency than those of CNL. For example, when we train the model on the MINIST database, the data traffic size and training latency can be respectively reduced by 69.63% and 81.18% by using the LFNL method. According to these results, we can conclude that the LFNL method can still achieve comparable testing accuracy, lower training latency, and less traffic compared with conventional centralized learning if the training data size is not large such as in the case of the MNIST and TIDIGITS datasets.

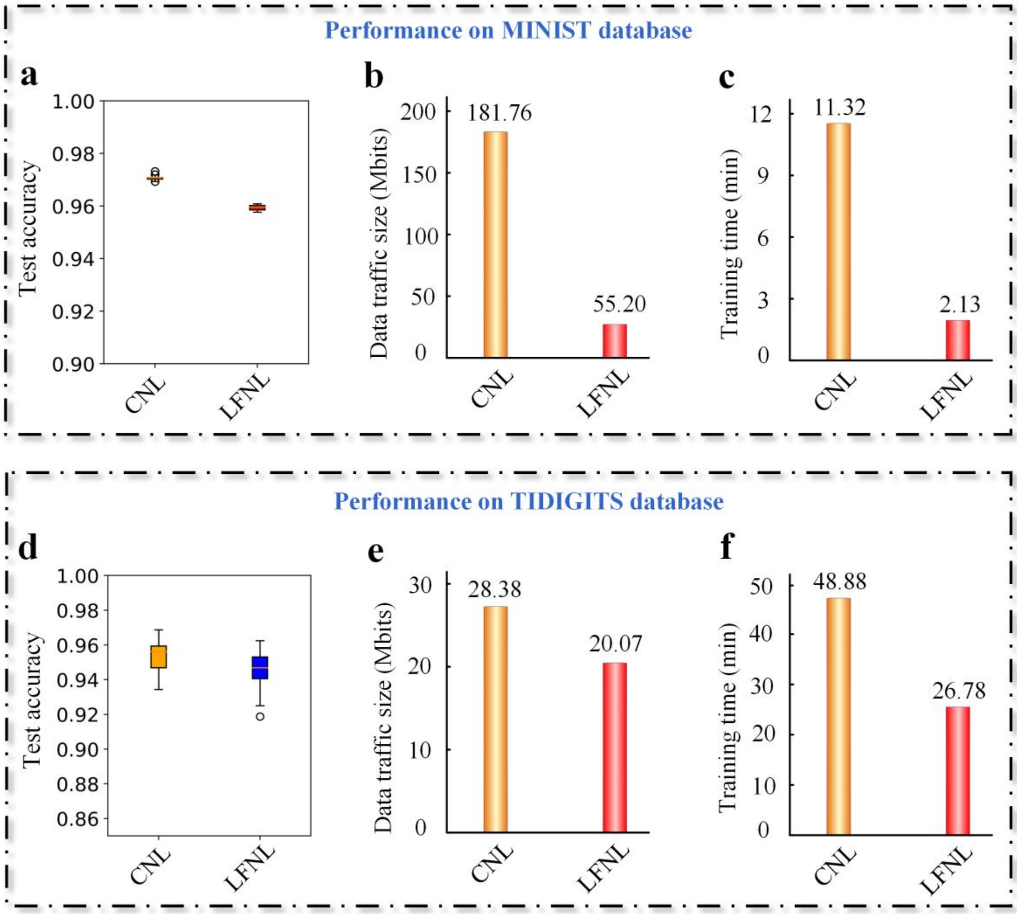

Supplementary Fig. 8: Classification evaluation on MNIST and TIDIGITS datasets. a-c, Classification accuracy, traffic size, and training latency performances of LFNL and CNL on the MNIST dataset, where we use 20% of the MNIST dataset for training. **d-f**, Classification accuracy, traffic size, and training latency performance of LFNL and CNL on the TIDIGITS dataset, where we use 30% of the TIDIGITS dataset for training.

Supplementary Note 6: Learning with noise added to clean raw datasets

We evaluate the classification accuracy change by adding Gaussian noise N (1, δ^2) to clean raw datasets. Here, we select the traffic sound dataset[15] as an example to evaluate the classification accuracy. Let P denotes the input signal power, and let δ^2 denote the added Gaussian noise power, then the signal-to-noise radio (SNR) is given by $SNR=10\log_{10}(P/\delta^2)$ in dB. From Supplementary Fig. R9, we can see that the classification accuracies of the two methods slightly decline as the SNR decreases when $SNR \geq 15$dB, because the added Gaussian noise power is not large in this region. However, when the SNR is lower than a certain level, i.e, $SNR<15$dB, the two methods suffer from a large classification accuracy decrease at the higher noise intensity. Across all noise intensities, the two methods have comparable classification accuracy performance and similar robustness.

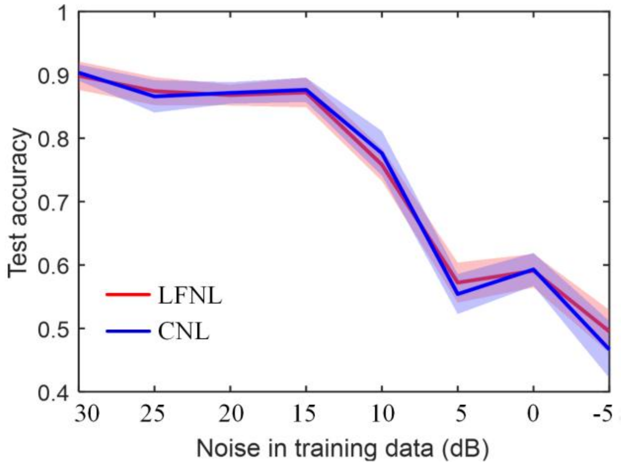

Supplementary Fig. R9: Performance change with respect to the additive Gaussian noise in the training data.

Supplementary Note 7: Learning with noise in model gradients

Now we consider the situation in which the gradients (or model parameters) may be obfuscated with added noise when the central server or the leader receives the gradients from edge devices. Here, the classification accuracy of LFNL and CFNL are evaluated with respect to the added noise in the gradients.We perform experiments using the VGG9 model on CIFAR10 and CIFAR100 datasets, where the 50000 32×32 RGB images of training data and 10000 32×32 RGB images of testing data were used. Similar to the work[18], Gaussian noise N (0, 1) multiplied by noise strength is added in the model gradients of all participating devices. From Supplementary Fig. 10, we can observe that the classification accuracy of the two methods degrades slightly as the noise strength increases. The results indicate that both

methods are robust to additive noise on the gradients. It is worth noting that LFNL still outperforms CFNL under different strengths of noise on the gradients.

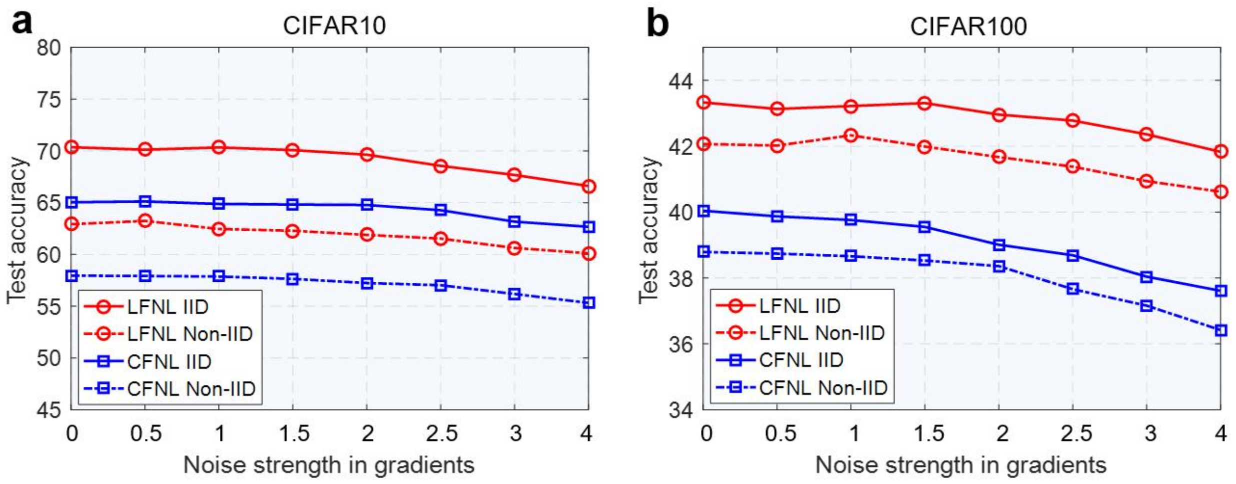

Supplementary Fig. 10: Impact of the gradient noise on the performance of LFNL vs CFNL when the training dataset is divided among 100 devices and 30 devices participate in each global round.

Supplementary Table 1: Network and training parameters for training on LFNL used to produce the experimental results in this work.

Parameters	Traffic sound	Traffic image	Radar gesture
Input size	128	1728	4800
Output size	3	3	5
Batch size	16	16	32
Optimizer	Adam	Adam	Adam
Adam parameter betas	(0.9, 0.999)	(0.9, 0.999)	(0.9, 0.999)
Adam parameter eps	10^{-6}	10^{-8}	10^{-8}
lr_scheduler	StepLR	StepLR	StepLR
lr_scheduler step size	50	15	15
lr_scheduler gamma	0.1	0.9	0.9
Learning rate	0.01	0.005	0.02

Supplementary References

1. Sjöberg A., Gustavsson E., Koppisetty A.C., & Jirstrand M. Federated learning of deep neural decision forests. machine learning, optimization, and data science. *Lecture Notes in Computer Science*, 11943 (2019).
2. Yang, Z., Chen, M., Saad, W., Hong C. & Shikh-Bahaei, M. Energy efficient federated learning over wireless communication networks. *IEEE Trans. Wireless Commun.* **20**, 1935–1949 (2021).
3. Yang, H., Zhao, J., Xiong, Z., Lam, K. -Y., Sun, S., & Xiao, L. Privacy-preserving federated learning for UAV-enabled networks: Learning-based joint scheduling and resource management. *IEEE J. Sel. Areas Commun.* **39**, 3144–3159 (2021).
4. Wen, D., Bennis, M., & Huang, K., Joint parameter-and-bandwidth allocation for improving the efficiency of partitioned edge learning. *IEEE Trans. Wireless Commun.* **68**, 2128–2142 (2020).
5. 3GPP TS 36.213, Evolved universal terrestrial radio access (EUTRA) physical layer procedures (Release 12), Sep. 2014.
6. Wu, F., Zhang, H., Di, B., Wu J. & Song, L. Device-to-device communications underlaying cellular networks: To use unlicensed spectrum or not? *IEEE Trans. Wireless Commun*. **67**, 6598–6611 (2019).
7. Chintamani, N., Yash, M., Nikhil, P., Vivek, P. & Sandeep, P. Smart traffic control using deep learning. Preprint at https://github.com/nikhilpatil99/Smart-Traffic-Management-Using-Deep -Learning (2019).
8. https://www.kaggle.com/hj23hw/pedestrian-augmented-traffic-light-dataset.
9. LeCun, Y., Bengio, Y. & Hinton, G. Deep learning. *Nature* **521**, 436–444 (2015).
10. Kaissis, G. A., Makowski, M. R., Rückert, D. & Braren, R. F. Secure, privacy-preserving and federated machine learning in medical imaging. *Nat. Mach. Intell*. **2**, 305–311 (2020).
11. Finlayson, S. G. et al. Adversarial attacks on medical machine learning. *Science* **363**, 1287–1289 (2019).
12. Han, T., et al. Breaking medical data sharing boundaries by using synthesized radiographs. *Sci. Adv. 6* eabb7973 (2020).
13. Ma, Z., Ma, J., Miao, Y., Liu, X., Choo, K. -K. & Deng, R. Pocket diagnosis: Secure federated learning against poisoning attack in the cloud. Preprint at https://arxiv.org/pdf/2009.10918.pdf (2009).
14. Cao, D., Chang, S., Lin, Z., Liu G. and Sun, D. Understanding distributed poisoning attack in federated learning. In *Proc. 2019 IEEE 25th International Conference on Parallel and Distributed Systems (ICPADS)*,233–239 (IEEE 2016).
15. https://www.kaggle.com/vishnu0399/emergency-vehicle-siren-sounds.
16. LeCun, Y., Bottou, L., Bengio, Y. & Haffner, P. Gradient-based learning applied to document recognition. *Proc. IEEE* **86**, 2278–2324 (1998).
17. Leonard, R. G. & Doddington, G. TIDIGITS LDC93S10. Web Download. Philadelphia: Linguistic Data Consortium (1993).
18. Venkatesha, Y., Kim, Y., Tassiulas, L. & Panda, P. Federated learning with spiking neural networks. *IEEE Trans. Signal Process.* **69**, 6183-6194 (2021).
